# Enriched Environment-Induced Neuroprotection against Cerebral Ischemia-Reperfusion Injury Might Be Mediated via Enhancing Autophagy Flux and Mitophagy Flux

**DOI:** 10.1155/2022/2396487

**Published:** 2022-06-27

**Authors:** Qi-Qi Zhang, Lu Luo, Mei-Xi Liu, Chuan-Jie Wang, Yi Wu, Ke-Wei Yu

**Affiliations:** ^1^Department of Rehabilitation Medicine, Huashan Hospital Affiliated to Fudan University, 200040 Shanghai, China; ^2^Department of Rehabilitation Medicine, Jinshan Hospital Affiliated to Fudan University, 201500 Shanghai, China

## Abstract

**Background:**

Enriched environment (EE) can protect the brain against damages caused by an ischemic stroke; however, the underlying mechanism remains elusive. Autophagy and mitochondria quality control are instrumental in the pathogenesis of ischemic stroke. In this study, we investigated whether and how autophagy and mitochondria quality control contribute to the protective effect of EE in the acute phase of cerebral ischemia–reperfusion injury.

**Methods:**

We exposed transient middle cerebral artery occlusion (tMCAO) mice to EE or standard condition (SC) for 7 days and then studied them for neurological deficits, autophagy and inflammation-related proteins, and mitochondrial morphology and function.

**Results:**

Compared to tMCAO mice in the SC group, those in the EE group showed fewer neurological deficits, relatively downregulated inflammation, higher LC3 expression, higher mitochondrial Parkin levels, higher mitochondrial fission factor dynamin-related protein-1 (Drp1) levels, lower p62 expression, and lower autophagy inhibitor mTOR expression. Furthermore, we found that the EE group showed a higher number of mitophagosomes and normal mitochondria, fewer mitolysosomes, and relatively increased mitochondrial membrane potential.

**Conclusion:**

These results suggested that EE enhances autophagy flux by inhibiting mTOR and enhances mitophagy flux via recruiting Drp1 and Parkin to eliminate dysfunctional mitochondria, which in turn inhibits inflammation and alleviates neurological deficits. *Limitations*. The specific mechanisms through which EE promotes autophagy and mitophagy and the signaling pathways that link them with inflammation need further study.

## 1. Introduction

Globally, numerous people die from or become disabled due to stroke every year [1]. Only a small proportion of stroke patients get effective intervention because of potential adverse reactions and the narrow time window for administering a treatment [2]. To reduce neurological impairments in stroke patients and improve their quality of life, feasible poststroke rehabilitation methods must be developed. Cerebral ischemia–reperfusion injury is a pathological process characterized by blood supply disruption, which leads to tissue hypoxia. The recovery of blood flow and tissue reoxygenation is frequently associated with tissue injury and severe inflammation [3].

Enriched environment (EE) is an effective rehabilitation intervention and includes various forms of neuronal stimulation, such as sensory stimulation, social stimulation, and exercise stimulation [4]. EE exposure has been shown to exert protective effects against the adverse effects associated with cerebral ischemia–reperfusion injury [5]; however, the underlying mechanisms remain elusive. Several studies have demonstrated that EE exposure can reduce the inflammatory response induced by cerebral ischemia–reperfusion injury and limit the extent of neuronal apoptosis [6–9]. Similar to apoptosis, autophagy is a type of programmed cell death; however, in contrast to the EE-mediated inhibition of neuronal apoptosis, EE reportedly promotes the occurrence of neuronal autophagy in acute stroke [10]. Autophagy reportedly dampens the effects of inflammation, thus playing a neuroprotective role [11]. Therefore, autophagy plays a protective role against acute stroke; however, the mechanisms by which EE promotes autophagy flux remain unclear. mTOR is a classical target for autophagy regulation [12]. Numerous studies have reported that autophagy is regulated via an mTOR-dependent pathway after stroke [13–15].

The interruption of cerebral blood flow in ischemic stroke leads to cerebral ischemia and hypoxia, causing dysfunction of mitochondrial oxidative phosphorylation and a series of subsequent ischemic cascade reactions [16]. Therefore, mitochondrial quality control is essential for reducing ischemic stroke-induced brain damage. In addition, mitochondrial dynamics and mitophagy participate in mitochondrial quality control. Increasing evidence has demonstrated that they contribute to the pathology of ischemic stroke and have been considered major therapeutic targets [17, 18]. Enhanced mitophagy can protect nerve cells from excessive damage caused by the accumulation of dysfunctional mitochondria [19]. In ischemic stroke, mitophagy is mainly mediated by the PTEN-induced putative kinase 1/Parkin signaling pathway [20]. Parkin can be recruited to damage mitochondria and induce mitophagy [21]. Excessive amounts of reactive oxygen species (ROS) can be produced by mitochondria after reperfusion [22], and ROS can induce mitochondrial fission [23]. Drp1 is a cytoplasmic GTPase that mediates mitochondrial fission [24]. Upon stress-induced mitochondrial damage, Drp1 is recruited to the mitochondria where it plays an important role in the mitophagy and energy response of the nervous system at baseline [24, 25]. There may be some connection between mitochondrial fission and mitochondrial autophagy. Moreover, mitochondrial fission occurs earlier than mitophagy [26].

In this study, we investigated whether EE plays a neuroprotective role by enhancing autophagy flux via the inhibition of mTOR and mitophagy flux through the recruitment of Drp1 and Parkin. We believe this is the first study to investigate the effect of EE on mitochondrial quality control in ischemic stroke and to verify whether EE regulates autophagy through the classical mTOR pathway.

## 2. Materials and Methods

### 2.1. Animals

A total of 105 C57BL/6 adult male mice (age, 8–12 weeks and weight, 24–28 g) were purchased from Zhejiang Vital River Laboratory Animal Technology Company. All animal experiments were performed in compliance with the National Institute of Health Guide for the Care and Use of Laboratory Animals; the experimental protocol was approved by the Institutional Animal Care and Use Committee of Fudan University, China (approval Nos. 2020 Huashan Hospital JS-163).

### 2.2. Surgery and Animal Groups

First, isoflurane anesthesia was induced in all mice (initial concentration 5%, maintained at 2%). The common carotid artery and external carotid artery were temporarily occluded, and the internal carotid artery was exposed and ligated. A nylon filament (Guangzhou Jialing Biotechnology Co., Ltd., serial number L2000) was introduced into the internal carotid artery through a small incision between the distal and proximal ends of the external carotid artery and preadded up to occlude the origin of the middle cerebral artery for 1 h. The filament was removed, and awake mice were placed in a cooling box. Regional cerebral blood flow (rCBF) in all tMCAO animals was monitored using laser Doppler flowmetry. Animals that died or failed to show ≥80% rCBF reduction relative to preischemic levels were excluded from further experiments. During the whole experiment, the room temperature was maintained at 25°C, and the mouse body temperature was sustained at 37°C. The tMCAO-treated mice were randomly divided into the following groups: tMCAO-treated mice maintained under standard conditions (tMCAO+standard condition (SC) group; *n* = 36) and tMCAO-treated mice maintained under EE conditions (tMCAO+EE group; *n* = 33). The remaining mice were subjected to a sham operation consisting of the ligation of the external carotid artery under isoflurane anesthesia, after which they were housed under standard conditions (sham+SC group; *n* = 36).

### 2.3. Housing Conditions

**Figure 1:**
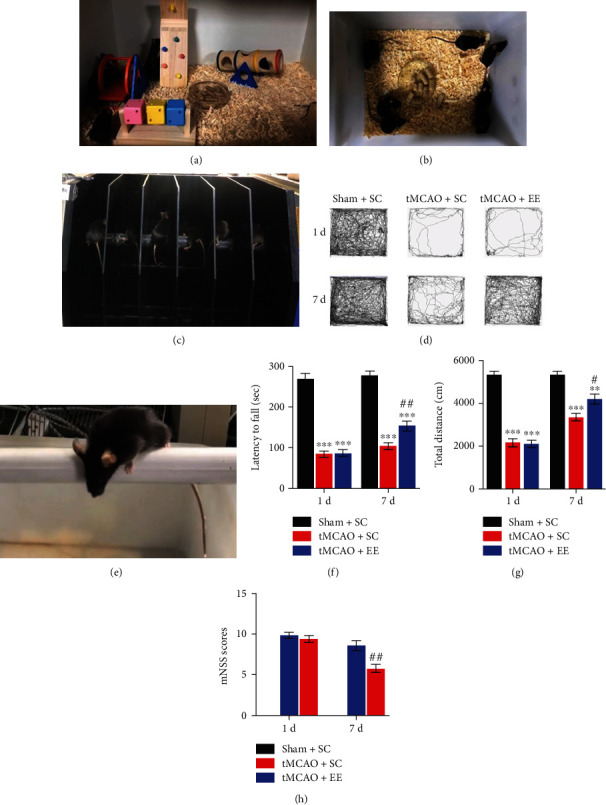
(a, b) Views of housing conditions. (a) Enriched environment (EE). (b) Standard condition (SC). (c–h) Behavioral tests, *n* = 6. (c, e) The experimental pictures of the rotarod test and mNSS performances, respectively; (d) the trajectory chart of the open-field test. (f–g) Statistical charts of behavioral tests. ^∗∗^*P* < 0.01 and ^∗∗∗^*P* < 0.001*vs*. sham+SC group; ^#^*P* < 0.05 and ^##^*P* < 0.01*vs*. tMCAO+SC group.

The EE group was placed in an 80 cm long × 60 cm wide × 40 cm high cage (Figure 1(a)), which was equipped with a fun room, a running wheel, a warped tube, a sports room, and colored blocks among other amenities to provide sensorimotor and cognitive stimulation. EE also provided enhanced social stimulation with 11 mice housed together. Mice were allowed to exercise voluntarily. Toys and toy placements were altered every three days to promote the novelty and exploration of EE. Mice in SC housing were placed in a standard cage (27 cm long × 21 cm wide × 16 cm high; Figure 1(b)) containing six mice per cage.

### 2.4. Behavioral Tests

The behavior of mice was tested 1 day and 7 days after the operation, respectively.

#### 2.4.1. Modified Neurological Severity Score (mNSS)

The mNSS is graded on a scale from 0 (no impairment) to 14 (severe impairment) and comprises three motor tests (tail raise, ambulation, and balance beam) and two sensory tests (pinna and corneal reflexes). A higher mNSS score reflects a more serious injury.

#### 2.4.2. Rotarod Test

Before the operation, mice were trained for three continuous days, and the test value obtained on the third day was used as the baseline. Only the mice that could remain on the rotating rod for at least 200 s were selected for the subsequent experiment. The total time for which the mice successfully remained on the rotating rod was recorded, and the mice that remained on the rod passively were recorded as having fallen. The rotarod accelerated from 4 to 40 rpm over a 300 s period.

#### 2.4.3. Open-Field Test

Mice were individually placed in four spontaneous activity boxes (each box: 25 cm long × 25 cm wide × 30 cm high) for 30 min daily for three days to adapt to the environment. The movement paths and distance traveled were recorded.

### 2.5. Histopathology

Seven days after the operation, mice were anesthetized with 1% pentobarbital sodium and perfused with precooled phosphate-buffered saline (PBS) via the heart and then treated with 4% paraformaldehyde for internal fixation. The brains were quickly removed and fixed with 4% paraformaldehyde for 48 h. These samples were then embedded in paraffin and cut into 5 *μ*m sections using a microtome; these sections were stained with hematoxylin and eosin and cresyl violet to observe any injury caused to the brain structure. We counted the surviving neurons per square micron according to the shape and number of Nissl bodies by ImageJ such that the density of Nissl bodies was the same as the density of surviving neurons.

### 2.6. Immunofluorescence Staining

Brain tissues (*n* = 6) were put into 30% sucrose solution for 24 h and cut into 30 *μ*m coronal sections using a Leica CM1950 cryostat (Leica Microsystems). After washing with PBS, coronal sections were sealed with 10% goat serum (C0265, Beyotime) and then incubated with primary antibodies of Drp1 (ab184247, Abcam) and Parkin (JF82-09, Novus). The sections were then sealed with 10% goat serum for 1 h and then incubated with Tomm20 antibody (H00009804-M01, Abnova). Thereafter, the sections were stained with fluorochrome-conjugated secondary antibody (ZF-0311, ZSGB-BIO), glial fibrillary acidic protein antibody (1 : 5000; ab7260, Abcam), and ionized calcium-binding adapter molecule 1 antibody (1 : 500; 019-19741, Wako). In brief, paraffin sections were immunostained with anti-nuclei (NeuN) antibody (ab177487, Abcam) at 4°C overnight and subsequently subjected to terminal deoxynucleotidyl transferase dUTP nick-end labeling staining using an In Situ Cell Death Detection kit (Cat. No. 11 684 795 910) according to the manufacturer's protocol. Finally, the sections were incubated with 4′,6-diamidino-2-phenylindole (C1002, Beyotime) nuclear dye, and the sections were visualized using a confocal laser scanning microscope (FV1000, Olympus). Sections from forebrain, midbrain, and hindbrain regions were randomly selected, and the ischemic penumbra was counted for statistical analysis.

### 2.7. Quantitative Real-Time–Polymerase Chain Reaction (qPCR)

Total RNA was extracted from the infarcted cortices of the three groups using an RNAprep Pure Tissue kit (DP431, Tiangen). Total RNA was reverse transcribed into cDNA with a FastKing RT kit (KR116, Tiangen). qPCR was performed using SuperReal PreMix Plus according to manufacturer's instructions (FP205, Tiangen). The primer sequences are listed in Table 1. All data were normalized against the levels of glyceraldehyde 3-phosphate dehydrogenase mRNA.

### 2.8. Western Blot

Total proteins of infarcted cortex were extracted using the protein extraction kit (BB-3101, Bestbio), and mitochondrial proteins of infarcted cortex were isolated using Tissue Mitochondria Isolation Kit (C3606, Beyotime). The concentration of proteins was measured using the bicinchoninic acid method, and loading buffer was added to obtain equal concentrations (3 *μ*g/*μ*L). Samples were subjected to 15% sodium dodecyl sulfate–polyacrylamide gel electrophoresis and transferred on a 0.45 *μ*m polyvinylidene difluoride membrane. After washing with tris-buffered saline with 0.1% Tween® 20, the membrane was sealed using a rapid sealing solution for 20 min and then incubated with primary antibodies at 4°C overnight, and the corresponding secondary antibodies (bs-0296G, Bioss) were incubated for 2 h at room temperature.

The following primary antibodies were used: LC3B (L7543, Sigma), p62 (18420-1-AP, Proteintech), mTOR (#2983, Cell Signaling Technology), p-mTOR (#5536, Cell Signaling Technology), IL-1*β* (16806-1-AP, Proteintech), IL-6 (#12153, Cell Signaling Technology), TNF-*α* (BS1857, Bioworld Technology) and *β*-actin (#3700, Cell Signaling Technology), COX IV (ab202554, Abcam), Drp1 (ab184247, Abcam), and Parkin (JF82-09, Novus).

### 2.9. Mitochondrial Membrane Potential Detection

Mitochondria were isolated using Tissue Mitochondria Isolation Kit and then immediately stained with a mitochondrial membrane potential (MMP) assay kit with JC-1 (C2006, Beyotime) and detected by flow cytometry.

### 2.10. Electron Microscopy

The mice were randomly selected from the three groups of mice, respectively, with 1% pentobarbital sodium anesthesia. After fixation with 2.5% glutaraldehyde, the brain was isolated, and peri-infarct areas were cut into 1 mm^3^ pieces, treated with 2.5% glutaraldehyde fixed solution overnight at 4°C, and then fixed with 1% osmium tetroxide. After double staining with citrate and uranium acetate, these pieces were cut into 70 nm ultrathin sections and observed using Hitachi HT7800 transmission electron microscope (Hitachi, Japan). The number of normal mitochondria, mitolysosomes, and mitophagosomes was counted from seven images per slide and three different slides for each mouse. Normal mitochondria are defined as having intact inner and outer membranes and neatly arranged cristae, as previously reported [27]. The mitophagosome has an obvious bilayer membrane structure, and the mitolysosome has a monolayer structure (the undecomposed mitochondrial membrane is not a complete membrane structure).

### 2.11. Statistical Analysis

Data were analyzed using GraphPad Prism 8.0 (GraphPad Software Inc., San Diego, CA, USA), and all results were expressed as means ± SEM. *P* values were calculated using two-tailed *t* test or Mann–Whitney test. A *P* value of <0.05 was considered statistically significant. The correlation between Drp1 and Parkin was analyzed using R language; *P* < 2.2*e* − 16 was considered indicative of a correlation between them.

## 3. Results

### 3.1. EE Exhibited Neuroprotective Effects in Neurological Function

We used a series of behavioral tests to assess the neurological function of mice (Figure 1). Compared with the sham+SC group mice, the tMCAO+SC group mice showed significantly impaired neurological function after stroke, which was demonstrated by an increase in mNSS (Figure 1(h)), a decrease in latency to fall (Figure 1(f)), and a reluctance to move and explore (Figure 1(g)). However, compared with the tMCAO+SC group mice, the tMCAO+EE group mice showed significantly improved neurological function (Figures 1(f)–1(h)).

### 3.2. EE Exhibited Neuroprotective Effects in Neurological Structure

**Figure 2:**
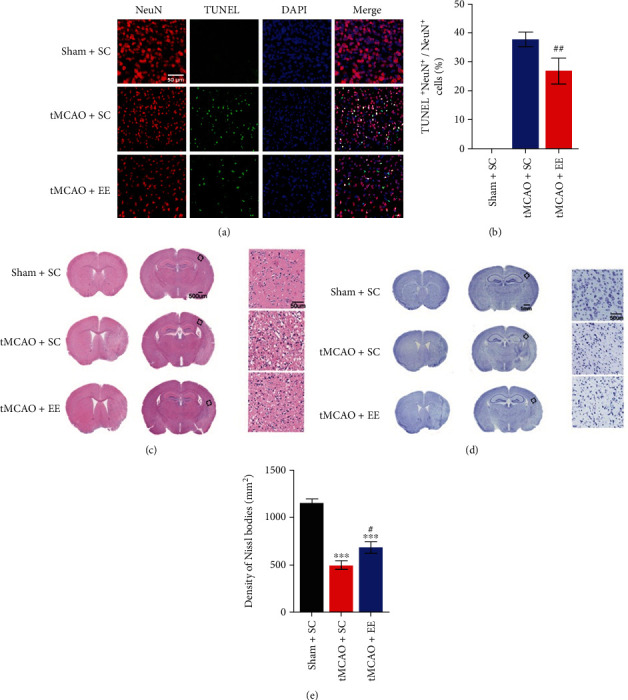
(a) TUNEL and NeuN staining results. (b) Comparison of apoptotic neurons in total neurons (apoptotic neurons in sham+SC group were not a part of the comparative study). (c) Hematoxylin and eosin staining results. (d) Cresyl violet staining results. The framed area is the ischemic penumbra. (e) Comparison of the density of cresyl violet bodies; ^∗∗∗^*P* < 0.001*vs*. sham+SC group; ^#^*P* < 0.05 and ^##^*P* < 0.01*vs*. tMCAO+SC group.

Next, we investigated the brain structure and neuronal apoptosis (Figure 2). No neuronal apoptosis was evident in the sham+SC group (Figure 2(b)). EE was found to significantly promote the recovery of brain structures after ischemic stroke (Figures 2(c)–2(e)). Moreover, EE reduced neuronal apoptosis in cerebral ischemia–reperfusion injury (Figures 2(a) and 2(b)).

### 3.3. EE Exhibited Neuroprotective Effects in Neurological Inflammation

**Figure 3:**
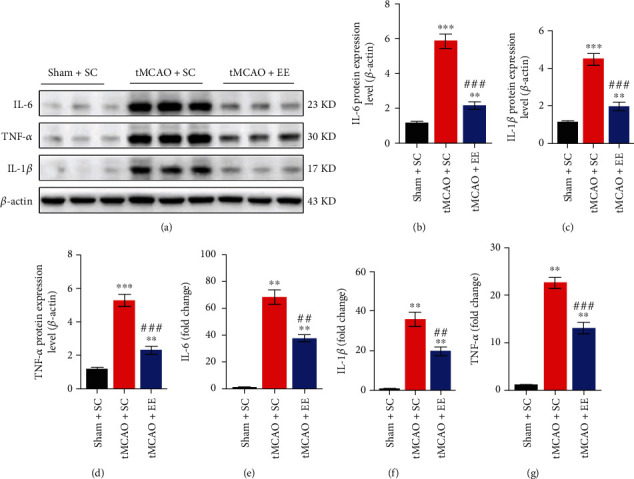
(a) Representative total protein Western blot images for inflammation. (b) Comparison of the *β*-actin-normalized IL-6 level between groups. (c) Comparison of the *β*-actin-normalized IL-1*β* protein level between groups. (d) Comparison of the *β*-actin-normalized TNF-*α* protein level between groups. (e–g) qPCR results. (e) Comparison of the changes in the IL-6 level between groups. (f) Comparison of changes in IL-1*β* levels between groups. (g) Comparison of changes in the TNF-*α* level between groups. ^∗∗^*P* < 0.01 and ^∗∗∗^*P* < 0.001*vs*. sham+SC group; ^#^*P* < 0.05, ^##^*P* < 0.01, and ^###^*P* < 0.001*vs*. tMCAO+SC group.

**Figure 4:**
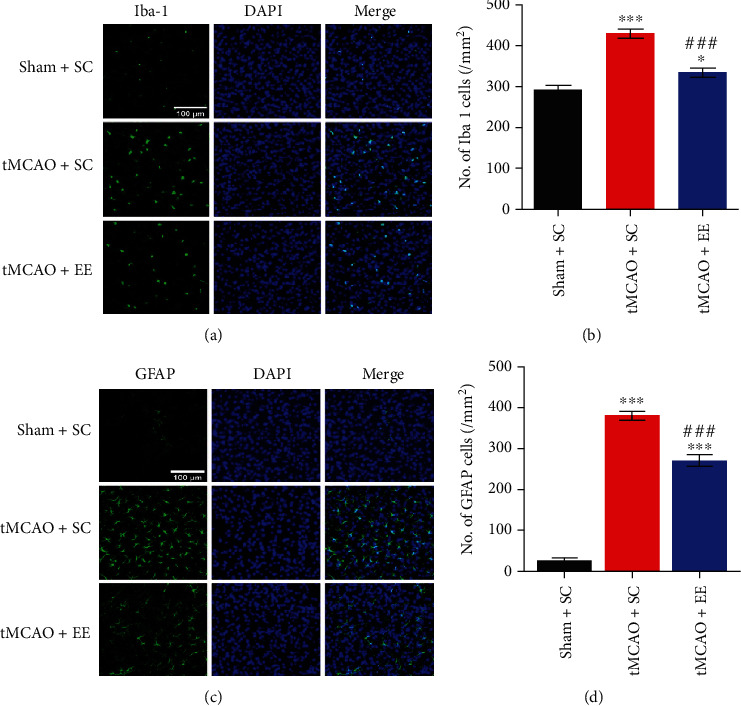
(a) Staining results for microglia. (b) Comparison of the number of microglia between groups. (c) Staining results for astrocytes. (d) Comparison of the number of astrocytes between groups (b, d) ^∗^*P* < 0.05 and ^∗∗∗^*P* < 0.001*vs*. sham+SC group; ^##^*P* < 0.01 and ^###^*P* < 0.001*vs*. tMCAO+SC group.

We also tested inflammation-related factors (Figures 3 and 4) and found that inflammatory reaction was evident after stroke. However, compared to the tMCAO+SC group, the tMCAO+EE group showed significantly decreased levels of reactive astrocytes, microglia (Figure 3), and inflammatory factors, including IL-1*β*, IL-6, and TNF-*α* (Figure 4).

### 3.4. EE Enhanced Autophagy Flux by Inhibiting mTOR

**Figure 5:**
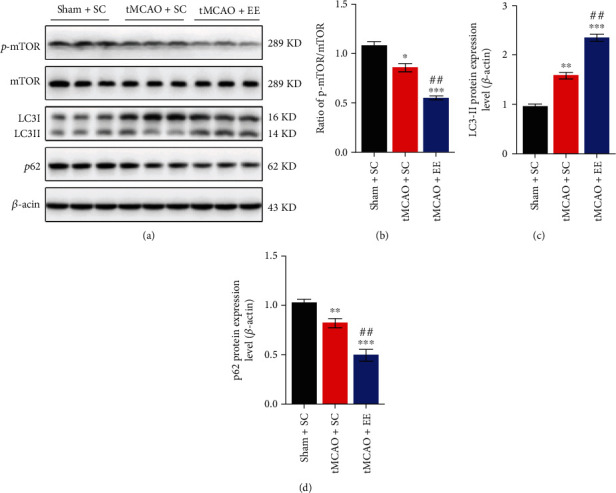
(a) Representative Western blot images for autophagy. (b) Comparison of the p-mTOR/mTOR ratio between groups. (c) Comparison of the *β*-actin-normalized LC3-II protein level between groups. (d) Comparison of the *β*-actin-normalized p62 protein level between groups. ^∗^*P* < 0.05, ^∗∗^*P* < 0.01, and ^∗∗∗^*P* < 0.001*vs*. sham+SC group; ^##^*P* < 0.01*vs*. tMCAO+SC group.

We determined the expression of total proteins that are related to autophagy. Compared to the sham+SC group, the other two groups showed more autophagy and suppressed mTOR expression after stroke (Figure 5). However, compared to the tMCAO+SC group, the tMCAO+EE group showed significantly higher LC3-II/*β*-actin ratio (Figures 5(a) and 5(c)) and significantly lower p62 levels (Figures 5(a) and 5(d)) and p-mTOR/mTOR ratio (Figures 5(a) and 5(b)). Taken together, these results suggested that EE enhanced autophagy flux by inhibiting mTOR.

### 3.5. EE Enhanced Mitophagy Flux via Recruiting Drp1 and Parkin

**Figure 6:**
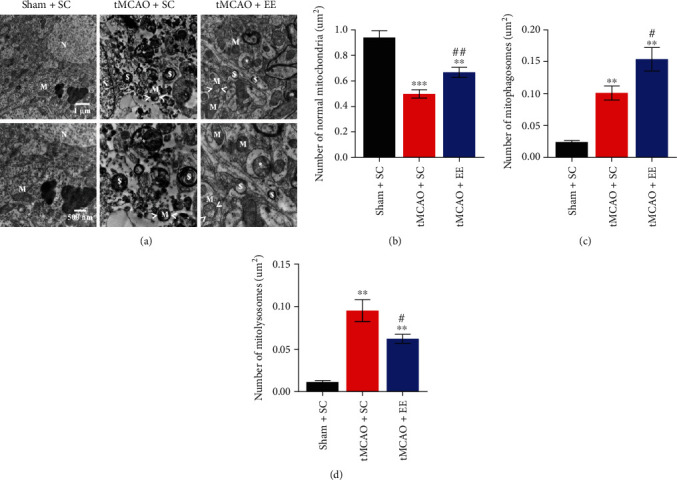
(a) The results of electron microscopy; N represents the nucleus, M represents normal mitochondria, > M < represents damaged mitochondria, ∗ represents mitophagosome with an evident bilayer membrane structure, and $ represents mitolysosome with monolayer membrane structure. (b) Comparison of normal mitochondria. (c) Comparison of mitophagosomes. (d) Comparison of mitolysosomes. ^∗∗^*P* < 0.01 and ^∗∗∗^*P* < 0.001*vs*. sham+SC group; ^#^*P* < 0.05 and ^##^*P* < 0.01*vs*. tMCAO+SC group.

**Figure 7:**
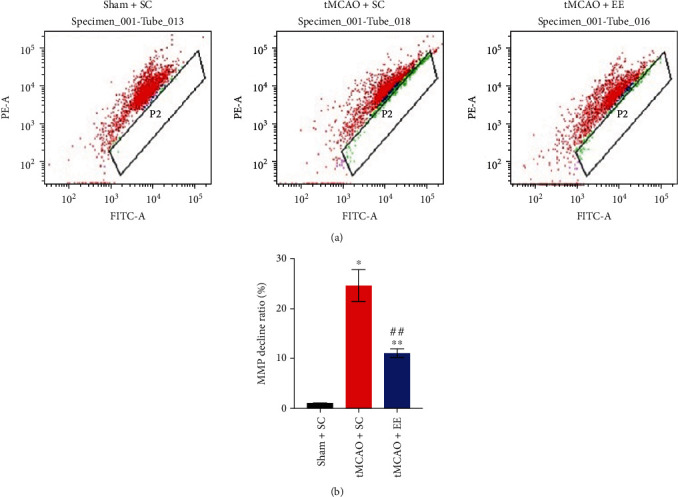
(a) The results of flow cytometry. (b) The decline ratio of mitochondrial membrane potential (MMP) calculated according to the ratio of the number of red and green mitochondria. ^∗^*P* < 0.05 and ^∗∗^*P* < 0.01*vs*. sham+SC group; ^##^*P* < 0.01*vs*. tMCAO+SC group.

**Figure 8:**
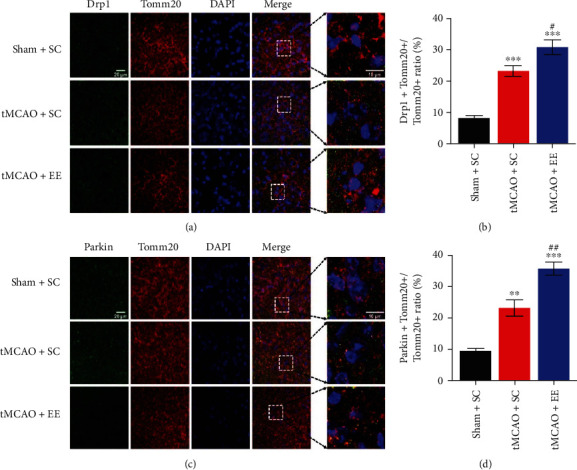
(a) The results of Drp1+Tomm20 costaining. (b) Comparison of the proportion of Drp1-positive mitochondria. (c) The results of Parkin+Tomm20 costaining. (d) Comparison of the proportion of Parkin-positive mitochondria. ^∗∗^*P* < 0.01 and ^∗∗∗^*P* < 0.001*vs*. sham+SC group; ^#^*P* < 0.05 and ^##^*P* < 0.01*vs*. tMCAO+SC group.

**Figure 9:**
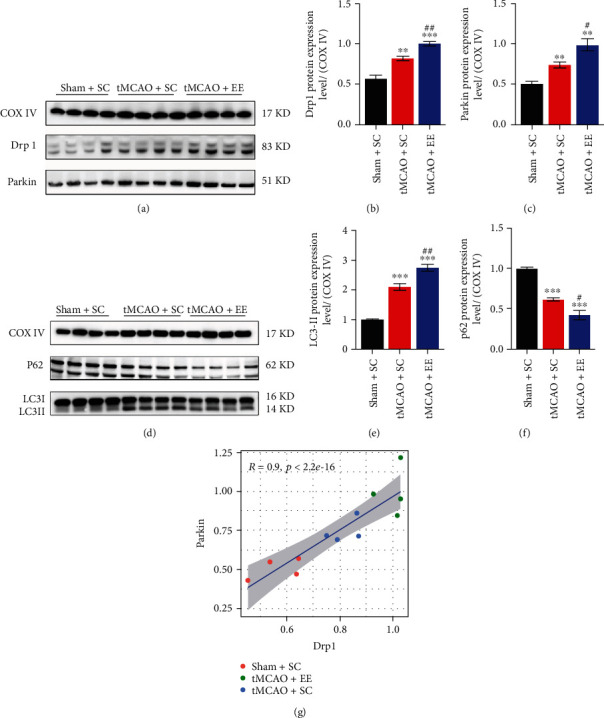
(a, d) Representative mitochondrial protein Western blot images. (b) Comparison of Drp1 protein expression in the mitochondria among the groups. (c) Comparison of Parkin protein expression in the mitochondria among the groups. (e) Comparison of LC3-II protein expression in the mitochondria among the groups. (f) Comparison of p62 protein expression in the mitochondria among the groups. ^∗∗^*P* < 0.01 and ^∗∗∗^*P* < 0.001*vs*. sham+SC group; ^#^*P* < 0.05 and ^##^*P* < 0.01*vs*. tMCAO+SC group. (g) The result of correlation analysis between Drp1 and Parkin; *P* < 2.2*e* − 16 and *R* = 0.9 indicate a highly positive correlation.

Next, we investigated the mitochondrial structure and function (Figures 6 and 7). It was obvious that the structure and function of mitochondria in the other two groups were damaged to varying degrees compared to the sham+SC group (Figures 6 and 7). However, compared to the tMCAO+SC group, the tMCAO+EE group showed more normal mitochondria and mitophagosomes and fewer mitolysosomes when observed under an electron microscope (Figure 6). We also observed that EE improved MMP (Figure 7). At the same time, we determined the expression of mitochondrial proteins (Figures 8 and 9). It was clear that the mitochondrial fission and mitophagy in the other two groups increased in varying degrees compared with the sham+SC group (Figures 8 and 9). As expected, the protein level of Parkin was significantly increased in the tMCAO+EE group compared to the tMCAO+SC group (Figures 8(c), 8(d), 9(a), and 9(c)), and we also detected increased LC3-II/COX IV ratio and reduced p62 expression in the tMCAO+EE group (Figures 9(d)–9(f)), indicating that EE could enhance mitophagy flux by promoting Parkin recruitment. Different from the tMCAO+EE group, the LC3 and p62 proteins of the tMCAO+SC group were accumulated, indicating that the mitophagy flux of the tMCAO+SC group was blocked (Figures 9(d)–9(f)). The tMCAO+EE group showed not only enhanced mitophagy but also increased mitochondrial fission by promoting Drp1 recruitment (Figures 8(a), 8(b), 9(a), and 9(b)). We also demonstrated a positive correlation between the levels of Drp1 and Parkin after ischemic stroke in the tMCAO+EE group (Figure 9(g)), suggesting that EE enhanced mitophagy flux via recruiting Drp1 and Parkin.

## 4. Discussion

Our study showed that EE could enhance autophagy flux by inhibiting mTOR and enhance mitophagy flux by recruiting Drp1 and Parkin; furthermore, it revealed the presence of a highly positive correlation between Drp1 and Parkin, which further inhibits the expression of inflammatory factors, thus resulting in an anti-inflammatory and neuroprotective effect. Our results showed that EE promotes autophagy through an mTOR-dependent pathway. However, as a comprehensive intervention, EE may promote autophagy in many ways, and it will be necessary to compare with mTOR gene knockout mice to verify whether autophagy can also be promoted by EE via an mTOR-independent pathway in further study. Our results suggest that mitophagy is blocked by cerebral ischemia–reperfusion injuries as well. Although ischemic stroke can increase the mitochondrial translocation of Drp1 and Parkin to some extent, the blockage of the downstream mitophagy flux leads to the accumulation of mitolysosomes and dysfunctional mitochondria. We decided in favor of counting normal mitochondria rather than damaged mitochondria because mildly damaged mitochondria may normalize via various repair mechanisms [28]. It is difficult to identify whether mitochondria are really completely damaged. More normal mitochondria also indicate fewer damaged mitochondria.

In this study, we provided evidence supporting the neuroprotective role of EE via autophagy. However, it is not clear how EE regulates autophagy through an mTOR-dependent pathway. The phosphoinositide 3-kinase- (PI3K)–protein kinase B (AKT) and mitogen-activated protein kinase (MAPK)/extracellular signal-regulated kinase (ERK)1/2 signaling pathways are known to upregulate mTOR activity, whereas AMP-activated protein kinase and sestrin can downregulate mTOR activity [29, 30]. EE may inhibit mTOR by inhibiting the PI3K–AKT and MAPK/ERK1/2 signaling pathways. The mechanisms through which EE activates autophagy to reduce inflammation remain unclear. Previous literature [31, 32] leads us to speculate that mTOR mediates these effects via some inflammatory regulatory factors, such as nuclear factor-kappa B (NF-*κ*B), which we will explore in future studies. EE reportedly downregulates the expression of inflammatory factors in neurons, astrocytes, and microglia through some inflammation-related pathways [33, 34]. In addition to the mTOR-dependent approach, autophagy could be mediated via an mTOR-independent approach as well, which may be associated with calcium overload and the activation of the Beclin-1 pathway [35, 36]. Moreover, EE-enhanced autophagy may occur in various brain cells, which may result in cell-specific effects. Only by identifying all of the factors and pathways involved can we obtain a better understanding of the role of autophagy in stroke.

Cerebral ischemia–reperfusion injury and mitochondrial damage are inseparable events as cerebral ischemia–reperfusion injury triggers a cascade reaction associated with the generation of ROS by damaged mitochondria [37]. Mitophagy can eliminate morbid mitochondria and reduce the production of ROS to alleviate inflammation [38]. Drp1 and Parkin are located in the cytoplasm [39, 40], and both of them are recruited to the mitochondria in response to mitochondrial injury, suggesting that some signal pathways in the cytoplasm, such as the PI3k/AKT pathway, may be involved in mitophagy after stroke [41]. Parkin is the upstream initiation molecule of the mitophagy process.

The inhibition of mitochondrial fission reportedly blocks Parkin-dependent mitophagy [42, 43]. This is consistent with our findings of the synchronous increase of Drp1 and Parkin in the tMCAO+EE group. Studies have shown that Parkin can promote mitophagy by recruiting Drp1 [44]. However, other studies have reported that Drp1 acts upstream of Parkin [45]. Parkin is an E3 ubiquitin ligase, which can inhibit mitochondrial fusion [46]. Briefly, mitochondrial fission is essential for Parkin-dependent mitophagy, and we believe that this may further encourage mitophagosome formation and subsequent lysosome capture with fragmented mitochondria. Genetic mouse models may be needed to further clarify the relationship between Drp1 and Parkin in EE-induced mitophagy. In cardiac ischemia-caused heart failure, mitophagy can be observed within 7 days, and the mitochondrial translocation of Drp1 matches the time of mitophagy [47]. However, the time of mitophagy and whether mitochondrial division and mitophagy are synchronized at different times after ischemic stroke remain unclear. Our current research only shows a highly positive correlation between mitochondrial division by Drp1and mitophagy induced by Parkin on the 7th day after ischemic stroke. Besides the Parkin-dependent mitophagy pathway, BNIP3 and FUNDC1 can also mediate mitophagy [48], and Drp1 also plays a role in Parkin-independent mitophagy pathway [49]. In addition to mitochondrial fission, mitochondrial fusion is also involved in the quality control of mitochondria [50]. Parkin inhibits mitochondrial fusion to facilitate mitochondrial fission, and thus, mitofusions may be involved in the subsequent structural and functional repair of mitochondria after the clearance of the majority of dysfunctional mitochondria.

In addition to mitophagy, autophagy of organelles, such as endoplasmic reticulum, is also instrumental in maintaining cell homeostasis [51]. Further studies of EE-induced organelle autophagy and cellular autophagy are required to clarify the neuroprotection mechanisms of EE in ischemic stroke.

However, our study also has some limitations. The specific mechanisms through which EE promotes autophagy and mitophagy and the signaling pathways that link them and inflammation remain unclear. Moreover, the relationship between autophagy and mitophagy and how they influence each other remain unknown. In further studies, in addition to elucidating the protective mechanisms associated with EE, we hope to identify new therapeutic targets for stroke and elucidate the pathophysiological processes that result in stroke-related injury.

Taken together, we confirmed the neuroprotective effects of EE in ischemic stroke and demonstrated the effect of EE on autophagy, mitochondrial dynamics, and mitophagy. Our findings are significant for the development of rehabilitation therapy.

## Figures and Tables

**Table 1:** Primers used for qPCR.

Target gene	Nucleotide sequence
*GAPDH*	Forward: AGGTCGGTGTGAACGGATTTG Reverse: GGGGTCGTTGATGGCAACA
*TNF-α*	Forward: GATCTCAAAGACAACCAACTAGTG Reverse: CTCCAGCTGGAAGACTCCTCCCAG
*IL-1β*	Forward: GCTGCTTCCAAACCTTTGAC Reverse: AGCTTCTCCACAGCCACAAT
*IL-6*	Forward: TAGTCCTTCCTACCCCAATTTCC Reverse: TTGGTCCTTAGCCACTCCTTC

## Data Availability

All data generated in current study are available from the corresponding author upon reasonable request.
